# Views of people living with dementia and their carers on their present and future: a qualitative study

**DOI:** 10.1186/s12904-023-01165-w

**Published:** 2023-04-10

**Authors:** Danielle Nimmons, Jill Manthorpe, Emily West, Greta Rait, Elizabeth L Sampson, Steve Iliffe, Nathan Davies

**Affiliations:** 1 Research Department of Primary Care and Population Health, Centre for Ageing and Population Studies, UCL, London, UK; 2 Social Care Workforce Research Unit, King’s College London, London, UK; 3 Division of Psychiatry, Marie Curie Palliative Care Research Department, Centre for Dementia Palliative Care Research, UCL, London, UK; 4 Barnet, Enfield and Haringey Mental Health Liaison Service, North Middlesex University Hospital NHS Trust, London, UK

**Keywords:** Dementia, Advance care planning, Social death theory

## Abstract

**Background:**

Dementia leads to multiple issues including difficulty in communication and increased need for care and support. Discussions about the future often happen late or never, partly due to reluctance or fear. In a sample of people living with dementia and carers, we explored their views and perceptions of living with the condition and their future.

**Methods:**

Semi-structured interviews were conducted in 2018-19 with 11 people living with dementia and six family members in England. Interviews were audio-recorded, transcribed and analysed using reflexive thematic analysis.

**Results:**

Findings were explored critically within the theory of social death and three themes were developed: (1) loss of physical and cognitive functions, (2) loss of social identity, and (3) social connectedness. Most participants living with dementia and carers wanted to discuss the present, rather than the future, believing a healthy lifestyle would prevent the condition from worsening. Those with dementia wanted to maintain control of their lives and demonstrated this by illustrating their independence. Care homes were often associated with death and loss of social identity. Participants used a range of metaphors to describe their dementia and the impact on their relationships and social networks.

**Conclusion:**

Focusing on maintaining social identity and connectedness as part of living well with dementia may assist professionals in undertaking advance care planning discussions.

**Supplementary Information:**

The online version contains supplementary material available at 10.1186/s12904-023-01165-w.

## Background

Dementia is the most common neurodegenerative condition worldwide and is estimated to affect around 47 million people [1]. In the early stages people living with dementia often experience early memory loss but as the condition progresses communication becomes more difficult, behaviour can change, walking becomes difficult and there is an increasing need for assistance with activities of daily living [1]. In the media and general society, the condition is often associated with negative images [2] and it is not uncommon for people to experience and be affected by stigma [3]. A UK survey found 62% of adults were worried about dementia in some way, and felt a diagnosis would mean their ‘life was over’ [4].

Many professionals see it as important that people living with dementia plan for the future through a process of advance care planning (ACP), particularly as the condition is unpredictable, with people experiencing a staggered decline with bouts of good and bad health, sometimes resulting in hospital admission [5]. In the UK there is a general professional consensus that opportunities for discussions about the future should be offered regularly (including around the time of diagnosis) but must be tailored to the individual, depending on their view of the future and readiness to discuss it [6]. This respects that people may not want to discuss the future due to fear, worry or denial [7] or other reasons, even though it means conversations about choices and preferences may not be possible, leaving it to family and/or professionals to make decisions.

Discussions may also not take place due to organisational challenges, such as a lack of access to specialist or primary care [8], professional inexperience or lack of confidence [9]. Although there is professional advice that ACP discussions should occur regularly, this does not always happen due to inequity in care provision [10]. Previous reviews of the literature have recommended that professionals hold several ACP discussions with people living with dementia over a longer period and involve family and significant others as early as possible, including when it is more difficult for the person living with dementia to communicate [11]. Despite challenges, it is important to discuss the future as ACP in dementia can lead to an increased number of advance directives and greater concordance between the person with dementia’s preferences and relatives’ decisions on their behalf [12, 13].

How people living with dementia view their futures is likely to vary. Some studies show negative views, for example, people may experience symptoms that leave them feeling unsure of themselves as the world becomes more unfamiliar [14]. While other studies have a more positive outlook, where people are not passive in how they experience the condition and use various strategies to cope with its challenges [15]. A recent Dutch study found those with early-stage dementia were willing to talk about their future, if given the opportunity [6]. They also felt it was important to live a meaningful life and maintain belongingness until end of life [6]. In recent years there has been an emphasis on framing the condition more positively and on people ‘living well’ with dementia [16], despite negatively held assumptions by the public [15]. Considering the varied views expressed in previous studies, the aim of our study was to explore, with an end-of-life focus, the views and perceptions of dementia and the future of people living with dementia in England.

## Methods

### Design

Qualitative study using semi-structured interviews, analysed using reflexive thematic analysis [17].

### Recruitment procedure

People living with dementia were purposively sampled from a range of sources including NHS memory services, general practice, carer or dementia organisations, and the National Institute for Health Research (NIHR) Join Dementia Research website. Potential participants from clinical services were identified and invited by a member of the clinical team. Interested and eligible participants either contacted the research team directly or with permission their details were passed to the research team. Recruitment invitations via non-clinical settings were sent by members of the research team or host organisation via email.

### Inclusion and exclusion criteria

People living with dementia were eligible if they met the following criteria:

- Clinical diagnosis of any type of dementia as categorised in ICD-10.
- Capacity to provide written informed consent to take part in an interview.
- Able to read and speak English.

Clinical teams checked eligibility, which included assessing if they felt potential participants had capacity. Experienced researchers trained in capacity assessment also assessed capacity when taking consent, at the start of the interviews and throughout. Those unable to provide informed written consent were not included. Family members were not recruited separately, only if the person with dementia wanted to be interviewed with them. Family members had to be adults, and able to consent to take part in an interview and to read and speak English.

### Data collection

Interviews were conducted by either a female research assistant or a male senior researcher, both of whom had training in qualitative research methods. Interviews were guided by an interview schedule which was informed by relevant literature [18–20], and supplemented with vignettes to prompt discussion [21]. The interview schedule can be seen in the Supplementary material along with an example vignette. The interview schedule was piloted with the study’s Patient and Public Involvement (PPI) group consisting of former carers, and with the first two participants, and revised. Interviews were conducted with the person with dementia alone, or with a family member if preferred by the participant living with dementia. Where a family member was present and took part in the interview they were consented, and their responses are included in the analysis. During dyadic interviews, participants living with dementia were asked questions first to ensure their views and experiences were heard. Carers were able to contribute when they wanted to but this was not the focus of the interview. All interviews were audio-recorded and transcribed verbatim. A research assistant checked all transcripts against the original audio files for any discrepancies and data were anonymised.

### Data analysis

Transcripts were imported into NVivo version 12 to facilitate analysis [22]. Reflexive thematic analysis was used to analyse data and develop themes [17]. One member of the research team (DN) coded all transcripts, while two others independently coded a further five. Through a series of discussions initial code lists were refined and initial themes developed by DN. Themes were then refined, while interpretation and meaning of each theme were discussed with the whole team (experienced in gerontology, primary care and palliative care), enabling them to contribute to findings and revise iteratively.

Following coding and the development of initial themes, we identified a similarity to some of the key themes within the concept of social death [23], a series of losses resulting in a disconnection from social life. Social death was first conceptualised in 1982 by Patterson in relation to slavery to describe how people can be considered unworthy and seen as dead when they are alive [24]. The theory has also been applied, at times controversially, to patients with chronic diseases, for example people living with dementia or, as it was then perceived, ‘suffering from dementia’ [23]. A recent concept analysis classified social death into three themes in relation to patients: the loss of social identity, loss of social relations, and deficiencies related to the inefficiency of the body and various diseases [25]. For example, as some chronic conditions advance, social roles change and people are unable to continue their previous social interactions as they are threatened by the body’s decline [23]. For some, losing social identity leads to exclusion or withdrawal from the wider community; associated with vulnerability, stigma and loss of physiological functioning [23]. In light of this, we shaped our findings and organised them within the three concepts underpinning the theory of social death to see if they offered an explanatory model. This enabled us to see if the theory of social death may offer insights into dementia experiences.

### Ethics approval

Health Research Authority (HRA) ethical approval was received (London Queen Square Research Ethics Committee (18/LO/0408) on 10.04.2018. Written informed consent was provided by all participants.

## Results

Eleven people living with dementia were recruited, all of whom had capacity. Six were dyadic interviews with a family carer and four were individual interviews. During dyadic interviews, all carers provided answers to all questions. Participant characteristics are presented in Table 1.

**Table 1:** Participant characteristics

	Person living with dementia N = 11	Family Carer N = 6
Age (years) *		
60–69	1	1
70–79	4	5
80–89	3	
90+	1	
Sex		
Male	6	1
Female	5	5
Ethnicity (census categories)*		
White British	7	4
White Irish	1	
East Asian	1	1
Mixed White and South Asian	1	1
Marital Status*		
Divorced	2	
Married/ Civil partnership	7	6
Single	1	
Age participant left education*		
At age 15/16	5	3
After age 20	5	3

*Not all participants provided this information

We arranged our themes under the three key concepts of social death theory [17], however we adapted the names of these concepts to reflect the participants’ words and the context of dementia: (1) loss of physical and cognitive functions, (2) loss of social identity, and (3) social connectedness. Table 2 provides an overview of the adapted concepts of the theory and our themes within them.

**Table 2:** Overview of concepts, themes and subthemes

**Loss of physical and cognitive functions** Theme 1: Staying present and the importance of maintaining self Theme 2: Discussing the future • Subtheme 2.1: Interview as enabler of advance care planning and discussions around the future • Subtheme 2.2: Planning for the future in discussions with family members
**Loss of Social Identity** Theme 1: Power, control and independence Theme 2: Perceptions of support and mixed perceptions of care homes
**Social Connectedness** Theme 1: Social view of dementia • Subtheme 1.1: Life legacy that can be maintained • Subtheme 1.2: Viewed by others and mixed metaphors of dementia and decline

## Loss of physical and cognitive functions

Across the interviews participants discussed their progressive decline, in particular the decline of their memory and cognitive functions. As discussed in the themes below, the loss of cognitive functions was often the focus of their discussion, rather than a change to physical functioning and abilities, although these often accompany advancing stages of dementia and indeed old age.

### Staying present and the importance of maintaining self

Many participants wanted to discuss the present and focus on what they were still able to do, rather than discussing the future and anticipated limitations. This discussion of the present appeared to either be a representation of their role and importance within their family, still demonstrating a sense of purpose or use to others, or more simply as a representation of things they enjoyed doing and as a way of reflecting they still enjoy life:

*“But I can still cook, can’t I? I can still wash up.“* Participant 03

*“But other than that, I still play golf three times a day – a week”*. Participant 02

Maintaining current levels of health and well-being was important to support their mood and some felt this was important to try to minimise the impact or delay the progression of their dementia:

*“I mean this morning I’ve done yoga. And we do it – [husband] and I do it every week. And I feel great when I’ve done it.*“ Participant 09

“*I still believe that at least I can delay and delay and delay it. ... And I’m still healthy, although I’ve got a heart condition.“* Participant 02

When asked about advanced dementia, many participants focussed on progressive memory loss which would lead to them relying on others such as their family. The narrative of dependence was often dominated by memory and less around physical function. Dementia was therefore seen as a condition of the mind (not the body) which would continue to deteriorate:

*“I’m not sure what dementia is, I don’t know what it means. I know it’s a side of the brain and the mind…I have knowledge confirmed of what’s going on in my mind or my brain.”* Participant 05

Participants believed that although their mind and memory will inevitably decline, by maintaining physical health they could still ‘get on’ with their lives and not be too restricted by the condition, for example, carrying out activities of daily living and ‘getting by’:

*“What is important to me really is to ensure that my health is, continues to be good physically, that I manage and that I can carry on – getting by in terms of memory”*. Participant 11

A few participants felt maintaining good physical health could help them ‘mask’ their dementia from other people, enabling them to still partake in enjoyable activities. As one participant said:

*“I think I handled it quite well because, as I say, there’s no, there’s no visible sort of way that gives away the fact that you’re suffering with a disease”*. Participant 10

This suggests this participant’s perception of dementia being a condition of the mind, instead of the body.

Masking their dementia was important to some participants who were worried about how others would treat or view them if they knew they had dementia. For example, one participant feared that others would point and talk about him if they could see that he had dementia:

*“People will think I will get more sensitive if you see me with that [dementia], and particularly for someone, you know, talking to each other, point out, pointing at me…but I’m afraid*.“ Participant 02

## Discussing the future

Discussing the future is discussed within two subthemes: ‘interviewer as enabler of discussion’ and ‘discussing with others and planning’.

### Interview as enabler of advance care planning and discussions around the future

Most participants did not want to discuss the future perhaps because they feared what it entailed, both in terms of losing their cognitive and physical functions, leading to increased need for support and care. They viewed dementia negatively and a condition that would only get worse*“[It is] Irreversible. One-way traffic…* “. However, for some this was as far as they wanted to initially go in discussing their dementia, with many diverting or closing down the conversation:

*“I don’t think about anything really about the future.*

**Okay, so have you… (interviewer)**

*I’m happy as I am.”* Participant 08

When asked directly about their future, most said they did not know what would lie ahead. However, as the interview progressed and rapport was built with the interviewer, many also recalled situations of people they knew approaching end of life. This included nursing a friend with terminal cancer, parents who had died in care homes, and friends with more advanced dementia:

***So you talked about maybe a need for a conversation now about diagnosis and things. What, as a family, do you understand about maybe the later stages of dementia? (interviewer)***

*”Not very much*”. Carer of Participant 03

When directly asked, this carer said she did not know what happens in the advanced stages of dementia, however she later discussed a friend whose husband with more advanced dementia was moving to a nursing home:

*“I’m getting a bit concerned this week because I have a friend in America with exactly the same problem with her husband. But I had a letter from her this week to say that he’s been taken into a home… through the memory clinic out there. And it’s all very, very distressing for her.“* Carer of Participant 03 (later)

These possible contradictions could suggest a degree of denial and that some carer participants perhaps knew more about later stages of dementia than they wanted to admit or felt uncomfortable talking about in the presence of their relative. Many carers seemed determined to focus on the present and not discuss the future, in part, due to fear and family experiences:

*“I think because he [Participant 07] has seen his mum in the home and ... they were lying in the bed all day and nobody came to look. So this is what we have seen in the home and that is in the mind.“* Carer of Participant 07

The interviews appeared at times to enable discussions around the future and advance care planning for participants and their carers, possibly providing them with one of the first opportunities to engage in these conversations either as an individual or with their partner who was being interviewed with them. However, responses to advance care planning questions were always short or vague:

**So if you were in that position, when you stopped eating, you’d stopped eating and drinking, and people – your family were trying to say, “No, no, you need to eat, you need to drink,” but you didn’t want to – what would you want them to think about in making that decision whether to stop or whether to think about those other options? (interviewer)**

*”Hmm, that’s difficult. I don’t know, if I didn’t know, I don’t know. If I didn’t know, I wouldn’t know, I’d have to wait and see what the problem was, you know, how I really, really felt*” Participant 08

This further indicates that participants did not want to talk about the future or were not ready to. This may have been for reasons such as fear, and/or the risk of causing sadness or distress for the person living with dementia. This often resulted in carer participants saying they would talk about these topics at some stage, without committing to a time:

*“I mean he gets a bit upset when you start talking about these sorts of things. But I mean I know it has got to be sought after, you know, later on, but I mean, at the moment, he copes very well”.* Carer of Participant 11

### Planning for the future in discussions with family members

Some participants living with dementia felt it was not worth talking about the future, when they would not be able to understand what was going on around them towards end of life:

“*I mean it has come to my mind, but I just try not to sort of dwell on it really. I just think I’ll take it as it comes. What’s the point of worrying about, you know, all those things that you worry about when you think about what’s going to happen when I die?“* Participant 09

This was despite some family carer participants showing interest in discussing the topic, while the participant living with dementia did not want to. This also illustrates how the interview enabled discussions around planning with family:

*“So, even I can picture this to what is lying ahead of us. But, yes, it would be nice to be prepared, or even he would like to – it will be good for him to know as well that what is there to look for”* Carer of Participant 07

This could be because several participants living with dementia openly said they would leave end of life decisions to their family carer when the time came, and some carers wanted to be prepared for this:

“*You know, I just think I’m sure my children will sort it out if they have to. I don’t have any great thoughts about it. You know, I try not to because I, you know, I want to enjoy what life that I’ve got without being miserable all the time*”. Participant 09

The default position adopted by participants living with dementia for their carers to make end of life decisions in a future when they no longer have capacity, is another example of how participants living with dementia wanted to stay in the present. This could be viewed as avoidant or a legitimate strategy. It also shows participants relinquishing more active or decision-making roles within their families, onto their children or partners.

By focusing on the present, maintaining current health and well-being, and showing a reluctance to discuss the future, participants living with dementia seemed to be deciding to leave discussions about the future to others while trying in the present to minimise further decline, although they knew their condition would worsen. In some cases, this was felt by interviewers to manifest as anger, as reflected in this participant’s response to his carer who said they had not discussed care homes or later stages of dementia:

*“No, this is the first time that it’s – we haven’t spoken about – because I didn’t think that I was about to pop my clogs (die).”* Participant 03

## Loss of social identity

Throughout the interviews there was an underlying discussion of social identity and how participants perceived and categorised themselves in relation to the progression of their dementia, other people and the world around them. Participants presented a narrative of independence and control to reduce the risk of social exclusion while the anticipated loss of independence was associated with advanced dementia and moving to care homes. We created the following themes within the category of loss of social identity: ‘Power, control and independence’ and ‘Perceptions on support and care homes’.

### Power, control and independence

Apart from planning for the future, participants living with dementia wanted to be in control of their lives, mostly in respect of everyday activities. Several participants felt the future was now out of their control, which worried them:

*“And people then getting worried about where they are and what they’re doing here and what’s going to happen next. And I’m not, I don’t have any control over it. I think it’s the feeling of lack of control that’s probably most worrying.”* Participant 04

However, control was also beginning to be affected by their current limitations. This included no longer being able to drive, cook or go out unaccompanied; activities that many people take for granted:

*“Well, you don’t have any choice. Once you’ve been diagnosed, they then say – “Hand in your [driving] licence*.” Participant 05

*“They told me, didn’t they, that it’s inadvisable to go out unaccompanied, I think, was the expression.”* Participant 03

Declining independence seemed to be altering parts of their identity. This was often viewed negatively by those living with dementia, as they felt independence was eroding which prevented them from living their lives as fully as previously. However, participants understood these changes were necessary to ensure their safety:

*“Well I can’t drive a car, which is another irritant. But I understand the necessity of stopping people like me driving, because we wouldn’t be safe on the road.”* Participant 10

To balance this, carers often focused positively on what their relative could do:

*“Well, you’re very independent, yes. You can do your shopping and you can read and you can eat, choose what you want to eat.”* Carer of Participant 06

However, many participants living with dementia were now needing assistance in many aspects of their lives, usually from family carers:

*“There’s very little I can do without her [wife] input, put it that way.”* Participant 05

Several acknowledged they would need more help from family as their dementia advanced. Those who did not have family felt they would have to employ people to help them:

*“No, I’ve really got no family that I can turn to. And, you know, they’ve moved on and I really, to some degree, now only got very minimal contact with my brother who lives in another part – he lives in [another part of UK]. So, so, so I would think that in terms of financial affairs, I’d have to employ someone specifically, you know, an accountant or someone to deal with that.”* Participant 11

For some participants with substantial resources these were not new arrangements:

*“We’ve got staff who look after the place. So you know, we’ve got a gardener, we’ve got a cleaner.”* Participant 11

Several participants mentioned role-reversal, where their spouse or children were carrying out the roles/jobs that previously held by the person with dementia. This included roles traditionally seen as signifying independence, such as managing one’s finances:

*“But you controlled your own money. I controlled mine and we both controlled ours.*

*But recently I’ve had to do it all for you, haven’t I...”* Carer of Participant 08

Again, many participants recognised that they could no longer carry out these duties and some recalled a time when they had held a more active role in the family:

*“But, you know, I’ve always been a leader and I don’t mean democratically. I’ve always looked upon the girls and the wives- I bought this house off my mother-in-law…I’ve never found a problem looking after the mother-in-law, both financially, and you know, like elderly ladies have to have baths etc. I never found it a problem,”* Participant 03

Not being able to carry out duties of responsibility was also seen as a loss of power by some:

*“What she [carer] said that I still want to hold power – in my mind I can correct a little bit what she just mentioned. And I try to hold on – but no, I’m very, very easy-going in my mind, because what can we do if I’m not able to make any decision?”* Participant 02

Another participant living with dementia discussed the situation of her mother who was also living with dementia. Evidently, she had arranged a personal alarm for her mother who did not wear it, as she considered it as a loss of independence:

*“So she does have an alarm that we pay for, but she refuses to wear it. So there’s many incidences of independence which are almost there in spite of what I’m trying to put in place to help to show how, how much independence you have, which is silly, because you need to wear the alarm.” Participant 06*

### Perceptions of support and mixed perceptions of care homes

Most participants viewed care homes very negatively and associated them with death:

*“That simply would be awful, a care home. Well I’d probably be dead. Most people in care home die, don’t they?”* Participant 06

Some participants said they would rather die or be dead than move to a care home:

*“I wouldn’t want that, yes. No, I’d sooner go and jump off a bridge.”* Participant 11

Care homes were seen as institutions where people are removed from their usual social settings, losing their social roles and therefore their social identity. Participants living with relatives felt they would lose social interaction in a care home and be left alone for long periods:

*“They [family] look after me. And I’ve company all the time, all the time. Then by the time, if I go to a home, then there won’t be company – in the morning, you are sleeping, if you’re there, you go to sleep and in the evening, you might get up – and by the time – well, if you get up or if you don’t, you go to sleep all the time, all the time.”* Participant 07

The care home environment was seen as important and one participant felt a home would not be able to cater to their needs or that they would not be able to relate to other residents:

*“Well, I don’t – I think the image of being in a place where I wouldn’t be able to – well, I wouldn’t be able to relate comfortably to the other people maybe. Or, if the place was going to be very basic and all the rest of it.”* Participant 11

However, two participants (P9 and C11) recalled positive care home experiences involving their relatives:

*“But I was very lucky to find two great homes for [mother] ”* Participant 09

Although, they admitted their relative had to pay for good quality care, for example, by using the assets raised by selling their home, as is generally the case in the UK:

*“And he went in with her – obviously he had to sell his house to pay, you know, help pay for it, you know, which is what happens with these now, these days, you know.”* Carer of Participant 11

Other participants were also more open to the idea of living in a care home when their dementia progressed, although all indicated they would prefer to live at home for as long as possible:

*“Yes, if the care home was like home, yes, I would be content to go there, but I can’t imagine them being as comfortable as – oh no, I mean the one that mum and dad was in, was very good.”* Participant 10

Timing of a care home move was also discussed, and all participants felt it would happen when the person living with dementia could no longer do things for themselves, family could no longer cope with caring for them, or they had lost capacity, although for some it would be a last resort:

*“*And to go into a care home would mess up the whole thing and be useless and I think the only thing to do is to die.” Participant 06

## Social connectedness

Participants described their previous and current social networks. These relationships and networks were constantly changing and were affected by factors, such as their past employment, and interactions with others from outside their circles of family or friends. Participants used a range of metaphors to help describe their dementia and its impact on their relationships and social networks.

### Social view of dementia: life legacy that can be maintained

Most participants reflected on their life, what it meant and how they did not want dementia to overshadow their achievements either academically, socially or just the person they had always been. Some referred to their successful working lives, for example in the military or public services. Some specifically mentioned how having a good memory had helped them at work, in contrast to the symptoms of their dementia:

*“I had a very, very, very good memory. I had a very good military career which I was, as a result of my memory and expertise*” Participant 03

They discussed the idea of loss, in particular loss of what they perceived as their identity. For example, one participant described this loss in relation to their previous occupation:

*“But, at that point, I was very, very aware that, you know, I was losing, really my vocabulary. That really bothered me, a big reader, works in libraries and so on. That was a big deal for me.”* Participant 01

Participants seemed to not want to be defined by their dementia and therefore focused on past careers or current roles. This was important for their social identity. For instance, one participant described current volunteer work, what that involved and how it benefitted them. They emphasised how some people living with dementia continue to add value to society despite their dementia:

*“I take a group of people round and talk about the history and about particular features of the cathedral. So that makes me, I do preparation the previous day to recall, because, after a month, for me, things slip away, so I just go through my prompt cards that I have about key facts and events. And the history of the cathedral. So that sort of refreshes the memory. I don’t read those when I go round, I do it all verbatim. So that’s that.”* Participant 11

### Social view of dementia: viewed by others and mixed metaphors of dementia and decline

Participants often used idioms, phrases or images through a series of different descriptions, and metaphors for describing their dementia and associated decline including:

*“Well, you’re not firing on all cylinders, you know. It doesn’t mean you’re totally gaga”* Participant 4

*“something that sort of invades the brain really, I think.”* Participant 09

However, some also used humour as a way of talking about their decline, possibly as a way of avoiding the reality of their condition:

*“Well if I got to the stage where I needed it, physical and mental stage – that’s never going to happen to me! Immortal.”* Participant 04

One participant described his family as a team, his daughters as ‘the managers’ and wife as ‘leader’:

*“I’m alright because we’re a team, you see. And [my wife] is the team leader.”* Participant 03

This participant was unable to carry out some activities, such as dealing with his finances or cooking. He relied on his family to do this and describing them as a team helped convey this. It seemed to be a way to help him cope with his gradual decline as he accepted the ‘team’ would have to do more for him and make more decisions for him as time passed. Being part of the ‘team’ also reflected his continued active involvement within his family and that he was not as dependent as he might be if he were outside the ‘team’, where he would have a less active role:

“*And I thought, “Oh gosh, I never want to get into a situation being old folk like that.” But it happens. But I’m sure I’m not going to do that to my team because they’re not going to allow me to do it.”* Participant 03

Many discussed how they were perceived by others and did not want to be recognised as having dementia. This was accomplished by not letting others know about their diagnosis, only close relatives and friends, or by hoping that by simply looking at them no one would know they had dementia:

*“I think I handled it quite well because, as I say, there’s no, there’s no visible sort of way that gives away the fact that you’re suffering with a disease.”* Participant 10

For some this meant not disclosing their dementia, so people would not treat them differently:

*“I thought I’m glad I haven’t told anybody, you know, because I mean it’s silly really, but, you know, I’ve kept – my friends say, when I’m taking the full, the full drug, you wouldn’t know, you’d never know. So I just wanted to keep it like that. I don’t want people doing things for me because they feel sorry for me.”* Participant 09

One participant described having dementia as wearing a mask but acknowledged that one day the mask will have to be peeled off. When asked about the later stages of dementia, he replied:

*“I think probably, nowadays, I think that I should be getting more happier, because by that time, probably I should give up – okay, I should peel off my mask.”* Participant 02

## Discussion

We conceptualised our findings into three concepts or categories, which we adapted from the Social Death theory [17], namely: (1) loss of physical and cognitive functions, (2) loss of social identity, and (3) social connectedness. Our participants living with dementia were focused on the present instead of thinking about and discussing their future and maintaining current levels of health was seen as a way to prevent deterioration. The interview seemed in many instances to enable discussions about the future, which was the subject raised in the study information material, this was particularly noted by interviewers in fieldnotes when interviewing dyads. However, even after enabling conversations many responses were vague, indicating a reluctance to discuss specifics or personal matters in detail during the interview. Overall dementia was viewed negatively and seen primarily as a condition affecting the mind. There was some suggestion that being in control of their physical body was more achievable at the present and could mediate their cognitive decline. Control seemed to help maintain independence while a lack of independence was associated with perceptions of advanced dementia, reliance on family, and care home moves. Our participants did not want dementia to overshadow past achievements or define who they were, and varied metaphors were used to describe the condition.

Social Death theory has previously been applied to people living with dementia. However, this theory no longer fits with how dementia is often viewed or understood in countries such as the UK as reflected in our findings; where there is more emphasis on capabilities and independence, instead of loss and social exclusion, and living well with dementia. Although helpful to consider Social Death in relation to our findings, we felt it was important to critically consider it and modify it, considering key important concepts of it as part of our analysis.

We explored how using the concept of social death could help reveal how participants living with dementia and their carers perceived the future of their condition and professional interactions. Social death was a concept used several years ago in respect of dementia by Sweeting and Gilhooly who argued that people who are treated as socially dead hold certain characteristics, including those with lengthy fatal illnesses, very old people, and people with a loss of personhood [26]. Some have argued that social death deprives people the dignity of a meaningful death [27]. However, it is also argued that loss of social identity, loss of connectedness and loss of physical abilities must be extreme for social death to be valid [23] (which our participants did not have as their dementia was early stage). Although, several participants held ideas about advanced and disabling dementia through the experiences of others [28], for example, family members who had dementia.

People living with dementia used to be described in a negative, dehumanised way as passive actors and socially dead [29]. However, as Kitwood and others have proposed, personhood and citizenship can be facilitated when people with dementia are seen and treated as socially active.[16] Also, empowerment and facilitation may help people living with dementia maintain their social identity and enable them to be part of a community [30], as emphasised in the policy ambitions of living well with dementia [31]. This is important as deterioration and a loss of personhood are not just due to the dementia itself but also affected by how people with dementia are treated by others, e.g. by being infantilised, in what is termed ‘malignant social psychology’ [16]. Being socially active may include people living with dementia taking part in discussions or choices about everyday life and taking their preferences into consideration [30]. This may be why all participants in our interviews focused on positive elements of their social identity, including maintaining independence and control of their lives. Also, it may explain why discussions around advance care planning were not raised, due to fear of upsetting the person living with dementia. Empowering them by following their wishes may have to include respecting their wishes not to explore the topic.

Metaphors reflecting active shaping of narratives were often used by participants to explain their dementia or how they were coping with the condition. This included describing an active presentation of donning a ‘mask’ to present a non-disabled status and presenting the family as a ‘team’ rather than overbearing. Patients often use metaphors to tell their stories and assigning meaning to their illness, which provides a bridge between the image of their old life and new one [32, 33]. For people living with dementia, choices of metaphors enable them to also preserve their social identity against negative cultural images of dependence, passivity and decline [33]. Metaphors with negative connotations are sometimes used by the media and scientific community to describe dementia, invoking fear in people [34], such as an ‘epidemic’ or ‘crisis’ [26]. People affected may be likened to victims and linked with a living death [34]. However, the use of metaphors in our participants’ narratives indicates more agency and autonomy in people living with dementia, enabling them to exercise voice and platform against the negative views of passivity and dependence associated with the condition [33].

Participants’ general reluctance to discuss the future and wish to live in the present links with previous research demonstrating these can be a barrier to advance care planning. A systematic review found patient factors such as ‘not being ready’, fear of death and denial were all reasons for lack of engagement, which sometimes could be explained in terms of coping [7]. This is also seen in other neurodegenerative conditions, such as Parkinson’s disease and multiple sclerosis, where it is unclear when is the optimal time to discuss the future and depends on similar patient factors [9, 35].

Participants living with dementia did not want the condition to define them or be part of their legacy, which could instead focus on their contributions to society. Post and colleagues have proposed that living a meaningful life is challenged by having dementia, as cognition is associated with maintaining self-control, independence, and relationships [36]. Demonstrating a meaningful legacy is therefore important in maintaining social identity in dementia and could be promoted by professionals. Social identity is also important in preventing social disenfranchisement, where people get labelled as a ‘dementia patient’, and so need to manage their condition as well as their identities [37]. The implications for practice are that healthcare professionals should elicit and consider patients’ identities and illness narratives, which may aid discussions around the future.

We found some carers seemed more open to discussing the future than the person living with dementia. This may be because they are witnessing their relative’s decline and are anticipating a potential crisis (when the person with dementia will be less able to make decisions) and that decisions will be needed, as described in other studies [38]. Participants living with dementia trusted their family carers to make decisions on their behalf about their future care, when the time comes. This finding confirms other studies [6], where a high level of trust and confidence was placed in family members taking on end of life decisions with professionals and advocating for their relatives’ wishes [39]. However, end of life discussions are difficult for some carers of people living with dementia and other conditions, as well as in other decision-making areas, such as legal-financial matters, care home moves, and making plans if the carer become unable to provide care [40]. Most of these topics were brought up during interviews, however, future care scenarios had not been fully discussed within the family in our view and this aligns with other research, where family carers report feeling unprepared when end of life care is needed [41].

Most participants viewed care homes negatively, fearing the isolation, loss of autonomy and dependency they associated with them. Some views were based on family experiences from some years ago. Care homes were viewed as institutions where, *de facto*, people are removed from their usual social settings, but also lose their social roles and therefore their social identity [23]. This confirms other research using social identity perspective theory that argues that care homes restrict social identity, as represented by a loss or giving up of possessions, clothing and activities, that represent a loss of independence and autonomy [42]. However, Paddock’s study included care home residents without dementia who shared a commonly held downward social comparison with those with dementia. Those residents who were more ‘cognitively superior’ than those with dementia felt their positive sense of identity was hindered because of the association of care homes with severe cognitive impairment [42].

However, a few participants whose relatives had positive experiences were more willing to consider moving to a care home in the future. There is an increasing body of international research highlighting the needs of people living with dementia from their own perspective, including those living in residential care [43]. This includes measures that can be taken to improve quality of life in care homes. For example, boredom is commonly experienced by residents with dementia and therefore organised activities are encouraged to combat this [43]. A feeling of restriction is also common, which can be counterbalanced by providing choice, while a homely environment and meaningful relationships can combat loneliness [43]. Funding of such improvements could lead to better care homes, promote social identity and connectedness; and help reduce the negativity associated with them.

### Strengths and limitations

This study explored the perspectives of people living with dementia, a group whose voices are often excluded from research and we explored a topic seldom researched. Our analysis was reflexive, our research team is diverse and from different disciplines including psychology, medicine, social work, and anthropology, which aided interpretation of results.

The study limitations should be noted. Only 11 people living with dementia were interviewed, which may not be representative of a wider population, however for this study we were much more guided by the concept of information power [44]. Information power encourages researchers to consider the richness of the dataset as opposed to the sample’s size and is recommended in recent discussions of thematic analysis [45]. No new themes were developed from the data during the eleventh interview, and there was strong, rich and clear dialogue between the researcher and participants. We therefore believe there was sufficient information power in this study and the sample size was adequate. People were excluded if they were not able to speak or read English, since we were not able to access interpreters, and further research should explore how findings might differ according to culture and ethnicity. Six of these interviews included a carer and this may have affected people’s frankness. A downside of dyadic interviews is that carers can ‘speak for’ or speak over the person living with dementia [41]. All those interviewed were able to provide written informed consent and we did not use communication aids or approaches to recruit participants with greater cognitive problems or other disabilities. Most of our sample was married and there were more men than women. Furthermore, socioeconomic data was not collected and differences according to income could not be analysed.

## Conclusions

We found participants living with dementia wanted to focus on the present and maintain control of their lives, while also maintaining their social identity and connectedness. We discussed key themes in relation to how people living with dementia and their carers view the future of their condition, in the context of social death theory and concluded that this needs to be used with caution in contemporary practice, policy making and professional interactions. Our participants were more empowered than this theory might suggest and wanted to present narratives of agency and social identity rather than ‘social death’. Focusing on this may allow for more open discussion around advance care planning to address the implications of decline as the condition progresses, if people so wish.

## Electronic supplementary material

Below is the link to the electronic supplementary material.

Supplementary Material 1

Supplementary Material 2

## Data Availability

Data sharing is not applicable to this article as no datasets were generated or analysed during the current study. If someone wants to request data, please contact Dr Nathan Davies (n.m.davies@ucl.ac.uk).

## Declarations

### Disclaimer

The authors are solely responsible for the content and not Alzheimer’s Society or NIHR.

## Abbreviations

ACP: Advance care planning
NIHR: National Institute for Health Research
PPI: Patient and Public Involvement
HRA: Health Research Authority
