# Supplementary material for: Views of people living with dementia and their carers on their present and future: a qualitative study
Source: BMC Palliat Care. 2023 Apr 10;22:38. doi: 10.1186/s12904-023-01165-w (PMC10084652; doi:10.1186/s12904-023-01165-w)
Supplement: Supplementary file 1 — Supplementary Material 1 [file 12904_2023_1165_MOESM1_ESM.docx]

**Interview Topic Guide – Persons with dementia**

**Part A) General question to explore or open up…**

1. What is your understanding of dementia?
   1. Symptoms
   2. How does it affect your life?
2. What is your understanding of the later stages of dementia?
3. Have you got any experience of someone else with dementia?
   1. Seen a friend or family member with dementia
   2. What do you understand about it from that experience?
4. Have you ever cared for someone at the end of life?
5. What is important to you now to have a good quality of life?
   1. Seeing family and friends, independence,
   2. How do you think this may change if your dementia were to become worse or your health were to decline?

----------------------------------------------------------------------------------------------------------------

**Part B) Exploration of planning and conversations already had**

1. Have you had discussions with those close to you about your diagnosis and future?
   1. What did these discussions include?
      1. More dependent and reliant on others
      2. Becoming ill and feeling unwell
   2. How do you approach these discussions?
2. Have you had discussions with professionals about your future care?
   1. What did these discussions include?
      1. Place of care
      2. DNAR
      3. Treatment and when to stop
      4. Lasting power of attorney
      5. Your will or funeral plans
   2. How do you approach these discussions?
   3. Who have you discussed this with? /who would you discus them with?
3. **Alternative to 5 and 6 –**
   1. **Are there any reasons why you have not had discussions about future care?**
   2. **How would you have discussions with family or professionals?**
   3. **How would you like these conversations to come about?**
      1. **Gradual or one off?**

----------------------------------------------------------------------------------------------------------------

**Part C) how things may change**

1. If your health were to deteriorate have you thought about what would be important to you?
   1. QOL
   2. Still included – making decisions or consulted
   3. Comfortable
   4. Still at home
2. What worries or fears do you have about your future health?
3. What would be important to you at the end of your life?
   1. Family
   2. Being at home
   3. Pain free
4. Are there any types of medical care or treatment you would not want to receive?
5. How could your family ensure these values are part of your end of life care?

----------------------------------------------------------------------------------------------------------------

**Part D) Exploration of decisions by others**

1. When would you want someone to make the decisions about your care?
   1. What decisions would you be happy for them to make?
      1. Medical/care
      2. Finance
      3. Every day – clothing/washing
      4. Do they make any now?
   2. Who would be involved in those decisions:
      1. Yourself?
      2. Family members/friends/other carers?
      3. Practitioners (incl. care home and care staff)?
      4. Anyone else? (faith minister, legal advisor, online resources such as Alzheimer’s Society)
2. How would you want them to be supported in making those decisions?
   1. Probe how this would involve them as the individual
   2. Decision aid probing

----------------------------------------------------------------------------------------------------------------

**Part E) Making decisions and the types of decisions**

We wanted to understand how you would approach some of these decisions and think out loud what you would be considering or want someone to consider.

1. Some everyday decisions:

- Leaving you alone at home for example to take the dog for a walk
- If you leave food and won’t eat
- If you want to sleep all day
- If you don’t want to get washed or dressed
- Ensuring you took your medication – crushing medication in food eg

1. Some more medical and symptoms based decisions. If you had difficulty with:

- If you appeared to be in pain or distress
- Going to the toilet
- Wandering or seeming to be agitated
- Talking to people
- *Consider asking more specific symptoms such as eating/swallowing difficulties, continuing or stopping treatment – if participant appears happy to discuss these.*

1. How would you approach thinking about:

- Engaging with practitioners/professionals – having conversations about care and the future
- Continuing treatment – antibiotics
- New carers or change in support – seeking help
- Where is the best place for you to be cared for?
- Planning forward to ensure you can remain at home? (if wanted)

1. What do you need to consider in these instances?
2. (TAILOR per topic) What would you want to happen if you become (agitated)?
3. What are the considerations/decisions that you need to be made here?
4. What are the right decisions? And how do you know?
5. How could these discussion be brought up/discussed?

Are there any other topics we have not discussed which you think are important?

----------------------------------------------------------------------------------------------------------------
