# Supplementary material for: Views of people living with dementia and their carers on their present and future: a qualitative study
Source: BMC Palliat Care. 2023 Apr 10;22:38. doi: 10.1186/s12904-023-01165-w (PMC10084652; doi:10.1186/s12904-023-01165-w)
Supplement: Supplementary file 2 — Supplementary Material 2 [file 12904_2023_1165_MOESM2_ESM.docx]

**MRS JONES**

Mrs Jones has now started hitting out at carers when they dress her. When the district nurse calls to dress her pressure sore, she becomes particularly aggressive and distressed. The district nurse tells her husband that ‘it is because she has dementia’. Her husband thinks that she may be in pain and she is not currently prescribed any pain killers.
